# Percutaneous transforaminal endoscopic decompression for the treatment of intraspinal tophaceous gout: A case report

**DOI:** 10.1097/MD.0000000000020125

**Published:** 2020-05-22

**Authors:** Xinji Chen, Guokang Xu, Qingfeng Hu, Tingxiao Zhao, Qing Bi, Yazeng Huang, Haiyu Shao, Jun Zhang

**Affiliations:** a Department of Orthopedics, Zhejiang Provincial People's Hospital; b Hangzhou Medical College People's Hospital, Hangzhou; c School of Clinical Medicine, Wenzhou Medical College, Wenzhou, Zhejiang; d Department of Orthopedics, Fuyang First People's Hospital; e Department of Orthopedics, The Affiliated Guang-Xing Hospital of Zhejiang TCM University, Hangzhou, Zhejiang; f Bengbu Medical College, Bengbu, Anhui, China.

**Keywords:** intraspinal tophaceous gout, percutaneous transforaminal endoscopic compression, surgical treatment

## Abstract

**Rationale:**

Intraspinal tophaceous gout is relatively rare condition presenting with major clinical manifestations, such as spinal cord or nerve roots compressions (radiculopathy). It is usually difficult to differentiate intraspinal tophaceous gout, lumbar disc herniation, stenosis of spinal canal, ossification of ligamentum flavum, and other degenerative spinal disorders from each other.

**Patient concerns:**

A 64-year-old man was admitted with a history of progressive low back pain for 11 months. He also presented with radiculopathy and numbness of his left lower extremity.

**Diagnoses:**

Preoperative computed tomography (CT) and magnetic resonance imaging (MRI) showed L4/5 disc herniation and lateral recess stenosis on the left side. During the operation of percutaneous transforaminal endoscopic decompression, intraspinal chalky white material was seen. Post-operative pathologic results confirmed the diagnosis of gouty tophi.

**Interventions:**

Percutaneous transforaminal endoscopic decompression was performed as treatment. Intraspinal chalky white material was seen. We removed most of the chalky white material and extruded nucleus.

**Outcomes:**

His symptom subsided rapidly and no deterioration was noted 1 year post-operatively.

**Lessons:**

Although intraspinal tophaceous gout is not commonly seen, clinicians should take it into consideration as a possible differential diagnosis when the patient exhibits axial pain or neurological deficits with risk factors of gout. We identified and treated this case with percutaneous transforaminal endoscopic decompression for the first time and got an excellent outcome. Percutaneous transforaminal endoscopic surgery proved to be an effective and minimally invasive alternative for identifying and treating intraspinal tophaceous gout.

## 1 Introduction

Tophaceous gout is a metabolic disorder caused by high serum uric acid, which mostly affects metatarsal-phalangeal joint, elbow, and knee.^[1]^ Pandya et al reported that the incidence of gout among American adults was about 4%.^[2]^ Due to the dietary changing in the past two decades, the prevalence of gout has increased about twofold.^[3–5]^ However, the involvement of gout in the spine is rarely reported, with only 133 relevant cases in literature.^[6]^ However, the incidence of intraspinal gout could be much higher than reported due to its insidious development and asymptomatic in most of the cases.^[6–8]^ So far, laminectomy through open approach has been reported as an effective treatment for those with neurologic deficits.^[9–12]^ Although with good outcomes in most of cases, laminectomy through open approach usually requires general anesthesia and large incision.

Endoscopic decompression for lumbar discectomy was introduced by Dr. Kambin and Dr. Gellman in 1983.^[13]^ With the development made by Dr. Yeung and Dr. Hoogland, it has been worldwide popularized among spine surgeons.^[14,15]^ With its merits of minimal invasiveness, safety and effectiveness, endoscopic decompression is one of the most widespread procedures for the treatment of multiple spinal disorders.^[16]^ With its visualization characteristic and magnify view, it is easier to identify rare lesion, such as intraspinal gout.^[17]^ Recently, percutaneous endoscopic surgery was successfully performed for cervical ligamentum flavum gouty tophus with good outcome.^[18]^ Here, we present the first case ever reported of symptomatic lumbar intraspinal tophaceous gout identified and treated with percutaneous transforaminal endoscopic decompression with good outcome.

### 1.1 Informed consent

The patient was informed about all aspects of the chosen treatment. Written informed consent was obtained from the patient for the treatment and subsequent submission for publication. In this case, a separate consent by Ethics Committee was not required.

## 2 Case report

### 2.1 Patient information

**Figure 1:**
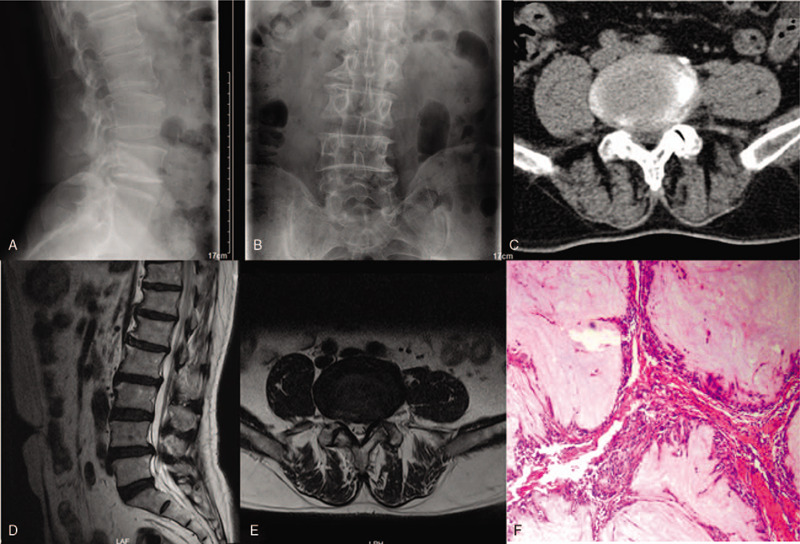
Lateral (A) and anterior-posterior (B) view showing mild degenerative lumbar scoliosis. Axial CT scan showing lateral recess stenosis on the left side at the level of L4/5 (C). Sagittal (D) and axial (E) view of MRI scan showing L4/5 disc herniation and lateral recess stenosis on the left side. Pathology examination revealing abundant deposited crystals surrounded by a foreign body-type giant cell reaction (F). H & E, 100×. CT = computed tomography.

A 64-year-old man was admitted with lumbar complaints and left sciatic pain for the last 11 months, with progressive worsening. He has no history of gout or spinal trauma. On neurological examination, we observed perineal and anterolateral left foot hypoesthesia, motor deficit during left foot flexion and extension, and left achillean hyporeflexia. Laseque sign was positive at 50°. Lateral and anterior-posterior radiographs showed degenerative features (Fig. 1A and B). CT scan and MRI revealed voluminous L4/5 disc herniation to the left side occupying great portion of the vertebral canal and stenosis of lateral recess (Fig. 1C–E). Laboratory examinations revealed that the serum uric acid was 324 μmol/L (normal range: 208–428 μmol/L), leukocytes count, erythrocyte sedimentation rate, and C-reactive protein were at normal range. Percutaneous transforaminal endoscopic surgery of L4/5 was performed for decompression.

### 2.2 Surgical procedure

The patient was placed prone with the lumbar mildly flexed on a radiolucent table. The procedure was usually conducted under local anesthesia with intravenous sedation. Under fluoroscopic guidance by C-arm, the operative field skin was marked and disinfected. The target point of initial needling is the posterior border of upper endplate of L5 and then replaced by a guidewire under biplane fluoroscopic control. A series of tapered obturators are inserted over the guidewire, a beveled working cannula is introduced over the obturators. After the obturators are withdrawn, an endoscope is inserted. We confirmed the correct position step by step using biplane fluoroscopic view.

**Figure 2:**
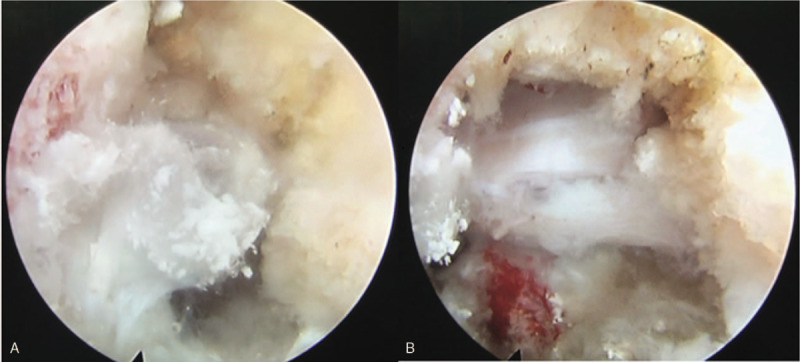
Intraoperative view (A) showed the chalky white material was occupying the epidural space and infiltrating the soft tissue. (B) After removing most of the chalky white material and the extruded nucleus, the transverse nerve was fully decompressed and could move with the heart rate and cough. CT = computed tomography, MRI = magnetic resonance imaging.

During operation, we removed part of the posterior longitudinal ligament, ligamentum flavum and the herniated disc to completely decompress the dural sac, traversing nerve and exiting nerve. Surprisingly, multiple amorphous chalky white lesions were unexpectedly identified occupying the epidural space around the extruded nucleus polposus and ruptured annulus fibrosis. This material was partially encapsulated by fibrous tissue and grossly infiltrated the bone and muscle (Fig. 2A). We removed the extruded nucleus polposus, as well as most of the chalky white lesions, till the freed dural sac and traversing nerve could move with the heart rate and cough (not with the breathing rate) (Fig. 2B). Free-floating dura mater in the irrigation fluid is a sign of sufficient decompression too. The white chalky material was submitted for hematoxylin and eosin sections. Pathological results demonstrated areas of an amorphous substance containing urate crystals surrounded by inflammatory cells and a multinucleated giant cell granuloma (Fig. 1F). Pathological examination confirmed the diagnosis of spinal gout. The patient confirmed the relief of radiculopathy before working channel and endoscope were evacuated. Surgical time was 1 hour and estimated blood loss was <50 mL.

### 2.3 Post-operation

The patient reported immediate relieve of radiculopathy of left lower extremity. No perioperative complication was occurred. With the guidance of rheumatologists, colchicine and febuxostat were prescribed to relieve the symptom and to lower the level of serum uric acid. No deterioration was found 1 year post-operatively.

## 3 Discussion

Although, no consensus was achieved yet for the treatment of intraspinal tophaceous gout, literatures suggested decompression of spinal cord and excision of the tophi for those patients suffered from progressive neurological deterioration or severe damage of joints.^[19,20]^ Kim et al recommended early surgical intervention to prevent further damage to the facet joints and neurological function, which could probably reduce the possibility of using spinal instrumentations.^[21]^

For patients with neurological deficits, open laminectomy with or without fusion used to be an effective treatment. Literatures demonstrated that laminectomy for patients with neurologic deficits caused by intraspinal tophaceous gout could achieve good outcomes in most of the cases.^[9–12]^ However, traditional laminectomy still has some shortfalls, such as general anesthesia, larger incision, longer operating time, more blood loss.

In order to minimize the trauma of surgery, as well as to reduce the operating time, surgical blood loss, hospital stay, complication rates, percutaneous transforaminal endoscopic decompression has been popularized worldwide in the last two decades. Moreover, with its magnified vision, endoscopic surgery could identify some rare lesions much easier than conventionally.^[22–25]^ Very recently, percutaneous endoscopic surgery was successfully performed for cervical ligamentum flavum gouty tophus with good outcome.^[18]^ Consistent with our present study, their experience demonstrated that percutaneous endoscopic technique could perform direct decompression with minimizing trauma and instability for the treatment of spinal gout.^[18]^ In the recent decade, percutaneous endoscopic surgery has been used worldwide used as an alternative and promising surgical alternative, with its advantages as reduced traumatization, better rehabilitation, and lower postoperative costs.^[16]^ Due to the shorter duration of surgery and local anesthesia required, it is better tolerated by elderly patients with morbidities.^[16]^

In this present study, we reported for the first time, how to identify and treat lumbar intraspinal tophaceous gout by percutaneous transforaminal endoscopic decompression. Since its first attempt in 1997, percutaneous transforaminal endoscopic decompression has been popularized worldwide for the treatment of spondylodiscitis, spinal stenosis, and other lesions.^[14]^ With the equivalent clinical results, it has advantages of shorter duration of surgery, less soft tissue trauma, better rehabilitation, cosmetics, and lower postoperative medical costs.^[14–16]^

It is proven as an effective and minimal invasive alternative for the treatment of intraspinal tophaceous gout. With its magnifying vision, usage of endoscopy could theoretically increase the incidence of identifying intraspinal tophaceous gout.

Recently, dual-energy CT (DECT) scanning has been used for the diagnosis of gout.^[26–29]^ Compared to CT scan, DECT allows substances composed with different chemical to appear distinct based on the different X-ray photon energy. So, DECT shows different color images of urate crystals and can be helpful to identify gouty tophi.^[26]^ A meta-analysis showed that DECT had high sensitivity 0.87 (0.79–0.93) (95% confidence interval [CI]) and specificity 0.84 (0.75–0.90) in the diagnosis of gout tophi.^[27]^ Moreover, doctors can measure the tophi's volume and make better treatment plan and prognosis with DECT.^[28]^ However, at present, DECT is not generally accessible because of its high cost, high radiation dose, uncomfortable noise and suboptimal evaluation of high body mass index (BMI) patients, which prevent its widespread use.^[29]^

Previous studies demonstrated that gout attacks are not always associated with the levels of serum uric acid. As a matter of fact, only a small part of patients with hyperuricemia finally develop gout. On the contrary, about 30% of patients with gout have normal serum uric acid levels.^[30–32]^ Our patient also showed a normal serum uric acid level of 324 μmol/L (normal range, 208–428 μmol/L). Intriguingly, Toprover reported that only 75.4% spinal gout patients had a history of gout or hyperuricemia.^[6]^

Although intraspinal tophaceous gout is rarely reported, it should be included in the differential diagnosis if the patient had severe back pain, even with serum uric acid in the normal range or without a history of gout. DECT is helpful and highly recommended for these patients. However, confirmation of the diagnosis is mostly based on the operation and pathological results. Percutaneous transforaminal endoscopic decompression is shown as an effective and minimal invasive alternative to better identifying and treatment of intraspinal tophaceous gout.

## 4 Conclusions

Although intraspinal tophaceous gout is relatively rare, it should be taken into consideration during differentiate diagnosis of degenerative lumbar disorders. DECT is highly recommended for diagnose; however, confirmation of the diagnosis is most possibly during the operation. Besides classical open surgery, endoscopic decompression is a novel alternative for the treatment of intraspinal gout with the merits of its minimal invasiveness, safety, and effectiveness.

## Acknowledgment

I thank Dr. Shem Singoyi for his assistance and thoughtful recommendations.

## Author contributions

**Conceptualization:** Xinji Chen, Guokang Xu, Qingfeng Hu, Jun Zhang.

**Data curation:** Xinji Chen, Guokang Xu, Qingfeng Hu, Tingxiao Zhao, Yazeng Huang, Haiyu Shao, Jun Zhang.

**Funding acquisition:** Jun Zhang.

**Methodology:** Tingxiao Zhao.

**Supervision:** Qing Bi, Yazeng Huang.

**Visualization:** Haiyu Shao.

**Writing – original draft:** Xinji Chen, Guokang Xu, Qingfeng Hu, Tingxiao Zhao, Jun Zhang.

**Writing – review & editing:** Qing Bi, Yazeng Huang, Haiyu Shao, Jun Zhang.
